# Intermedin in the Paraventricular Nucleus Attenuates Cardiac Sympathetic Afferent Reflex in Chronic Heart Failure Rats

**DOI:** 10.1371/journal.pone.0094234

**Published:** 2014-04-07

**Authors:** Xian-Bing Gan, Hai-Jian Sun, Dan Chen, Ling-Li Zhang, Hong Zhou, Li-Yan Chen, Ye-Bo Zhou

**Affiliations:** 1 Key Laboratory of Cardiovascular Disease and Molecular Intervention, Department of Physiology, Nanjing Medical University, Nanjing, China; 2 Department of Physiology, Anhui University of Chinese Medicine, Hefei, China; 3 Laboratory Center for Basic Medical Sciences, Department of Medical Physiology and Biochemistry, Nanjing Medical University, Nanjing, China; 4 Department of Haematology, the 2nd Affiliated Hospital of Harbin Medical University, Harbin, China; Max-Delbrück Center for Molecular Medicine (MDC), Germany

## Abstract

**Background and Aim:**

Intermedin (IMD) is a member of calcitonin/calcitonin gene-related peptide (CGRP) family together with adrenomedullin (AM) and amylin. It has a wide distribution in the central nervous system (CNS) especially in hypothalamic paraventricular nucleus (PVN). Cardiac sympathetic afferent reflex (CSAR) is enhanced in chronic heart failure (CHF) rats. The aim of this study is to determine the effect of IMD in the PVN on CSAR and its related mechanisms in CHF rats.

**Methodology/Principal Findings:**

Rats were subjected to left descending coronary artery ligation to induce CHF or sham-operation (Sham). Renal sympathetic nerve activity (RSNA), mean arterial pressure (MAP) and heart rate (HR) were recorded. CSAR was evaluated by the RSNA and MAP responses to epicardial application of capsaicin. Acute experiments were carried out 8 weeks after coronary ligation or sham surgery under anesthesia. IMD and angiotensin II (Ang II) levels in the PVN were up-regulated in CHF rats. Bilateral PVN microinjection of IMD caused greater decreases in CSAR and the baseline RSNA and MAP in CHF rats than those in Sham rats. The decrease of CSAR caused by IMD was prevented by pretreatment with AM receptor antagonist AM22-52, but not CGRP receptor antagonist CGRP8-37. Ang II in the PVN significantly enhanced CSAR and superoxide anions level, which was inhibited by PVN pretreatment with IMD or tempol (a superoxide anions scavenger) in Sham and CHF rats.

**Conclusion:**

IMD in the PVN inhibits CSAR via AM receptor, and attenuates the effects of Ang II on CSAR and superoxide anions level in CHF rats. PVN superoxide anions involve in the effect of IMD on attenuating Ang II-induced CSAR response.

## Introduction

Cardiac sympathetic afferent reflex (CSAR), a sympatho-excitatory reflex, can be induced by stimulation of cardiac sympathetic afferents with exogenous or endogenous chemicals from myocardium during myocardial ischemia [1], [2]. Chronic heart failure (CHF) is accompanied by the enhanced sympathetic nerve activity (SNA) [3]–[5], and the enhanced CSAR partially contributes to the sympathetic activation in CHF rats [5]–[7]. Suppression of sympatho-excitation has been considered as a strategy in treating patients with CHF [8], [9], so the inhibition of CSAR may be a good target for decreasing sympathetic activation in CHF.

Intermedin (IMD) is widely distributed in peripheral organs and central nervous system (CNS) and belongs to calcitonin/calcitonin gene-related peptide (CGRP) family together with adrenomedullin (AM) and amylin [10]–[13]. IMD, CGRP and AM share the receptor system including calcitonin receptor-like receptor (CRLR) and receptor activity-modifying proteins (RAMPs). The CRLR/RAMP1 complex forms the CGRP receptor, whereas CRLR/RAMP2 or CRLR/RAMP3 complex forms the AM receptor [10]. In the present, no unique receptor has been identified for IMD, but it can non-selectively bind to all three CRLR/RAMP complexes. CRLR and RAMPs have been found in the paraventricular nucleus (PVN) of hypothalamus, and there is abundant IMD-like immunoreactivity in the PVN including both parvocellular and magnocellular cells [11], [14]–[16].

PVN is an integrative site in regulating sympathetic outflow and cardiovascular activity [17], [18], and it is also a component of central neurocircuitry of the CSAR [19], [20]. Previous studies have shown that PVN is involved in excessive sympathetic activation and enhanced CSAR in CHF [21]–[25]. Angiotensin II (Ang II), AT1 receptors and reactive oxygen species (ROS) in the PVN play an important role in the central modulation of CSAR, contribute to the pathogenesis of enhanced CSAR in CHF [23]–[25], and AT1 receptor mRNA antisense normalizes the enhanced CSAR in CHF rats [26]. IMD has different effects on SNA depending on where it is applied in the brain. Administration of IMD into the lateral cerebroventricle or NTS increased SNA [27]–[29], but we recently found that IMD in the PVN attenuated sympathetic activity [30] and CSAR (data not published) in hypertensive Rats. It is interesting to know the roles and mechanisms of IMD in the PVN in CSAR in CHF rats because of the importance of the PVN in the pathogenesis of CHF.

Ang II in the PVN promotes the enhanced CSAR in rats with CHF [25], [31], [32], and the decrease of endogenous Ang II by angiotensin converting enzyme inhibitor captopril in the PVN normalizes the enhanced CSAR in rats with CHF [24]. Ang II in the PVN increases superoxide anions level which mediates CSAR and the effect of Ang II in the PVN on CSAR in CHF rats [23]. Ang II exposure induces remarkable increases in the expression of endogenous IMD and its receptor components in H9c2 cell cultures [33]. IMD exerts an antihypertrophic effect caused by Ang II on neonatal cardiomyocytes by reducing the level of superoxide [34]. In many animal disease models, IMD has a protective effects on some tissues or cells via the inhibition of oxidative stress [35]–[40]. It is not known whether PVN superoxide anions involve in the effect of IMD on attenuating Ang II-induced CSAR response. Therefore, the present study was designed to determine whether IMD in the PVN inhibits SNA and CSAR and whether IMD in the PVN attenuates Ang II-induced CSAR response and its related mechanisms in CHF rats.

## Methods

### Animals

Experiments were carried out on male Sprague-Dawley rats weighing 300–400 g. The procedures were approved by the Experimental Animal Care and Use Committee of Nanjing Medical University and complied with the Guide for the Care and Use of Laboratory Animals (the 8th edition, 2011). The rats were maintained on a cycle of 12 h light and 12 h darkness in a temperature-controlled room and provided free access to laboratory chow and water.

### CHF model

CHF was induced by coronary artery ligation method as previously described [5]. Briefly, rats were anesthetized with sodium pentobarbital (60 mg/kg, i.p.) and subjected to the ligation of the left anterior descending coronary artery or sham operation using sterile techniques. Sham-operated rats (Sham) received similar surgery except their coronary arteries were not ligated. The criterion for CHF was that left ventricle end-diastolic pressure (LVEDP) was higher than 12 mmHg. At the end of experiment, infarct size was expressed as a percentage of the left ventricle (LV) surface area [26].

### General procedures of acute experiment

Acute experiment was carried out 8 weeks after surgery. Each rat was anaesthetized with intraperitoneal injection of urethane (800 mg/kg) and a-chloralose (40 mg/kg). A midline incision in the neck was made to expose trachea and carotid artery. The trachea was intubated and connected to a rodent ventilator (Model 683, Harvard Apparatus Inc, Holliston, MA, USA) for mechanical ventilation. A pressure transducer was used via a catheter in the right carotid artery for the measurement of mean arterial pressure (MAP). The MAP and renal sympathetic nerve activity (RSNA) were simultaneously recorded with a PowerLab data acquisition system (8/35, AD Instruments, Castle Hill, Australia). Supplemental doses of anesthetic agents were applied to maintain an adequate depth of anesthesia during the experiments.

### RSNA recording

A retroperitoneal incision was made for isolation of the left renal sympathetic nerve. The nerve was cut distally to eliminate its afferent activity and placed on a pair of silver electrodes which were immersed in warm mineral oil. The RSNA was amplified with a four channel AC/DC differential amplifier (DP-304, Warner Instruments, Hamden, CT, USA) with a high pass filter at 100 Hz and a low pass filter at 3,000 Hz. The RSNA was integrated at a time constant of 100 ms. The background noise was determined as previously reported [41].

### Evaluation of CSAR

The rats used to evaluate the CSAR were subjected to vagotomy and baroreceptor denervation [23]. To expose the heart, a limited left lateral thoracotomy was performed and then the pericardium was removed. The CSAR was induced by stimulating cardiac sympathetic afferents with a piece of filter paper (3 mm×3 mm) containing capsaicin (Cap, 1.0 nmol in 2.0 μl) on the non-infarct area of the left ventricle. The filter paper was removed 1 minute later, and the ventricular surface was rinsed three times with normal saline at 37°C. The CSAR was evaluated by the RSNA and MAP responses to the epicardial application of Cap [23].

### PVN microinjection

**Figure 1:**
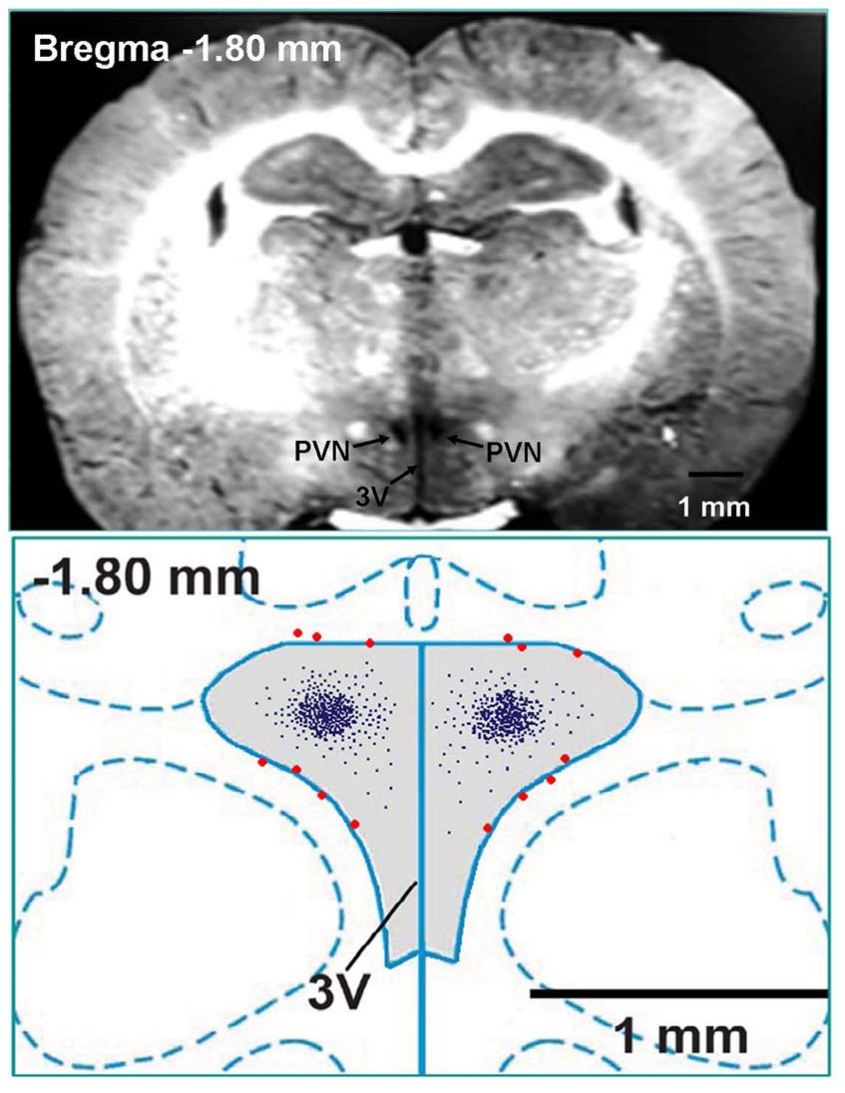
Microinjection sites in PVN region. Upper panel, a representative photo of microinjection sites in the PVN evaluated by 50: paraventricular nucleus of hypothalamus; 3V: the third ventricle.

The stereotaxic coordinates for the PVN were 1.8 mm caudal from bregma, 0.4 mm lateral to the midline and 7.9 mm ventral to the dorsal surface using a stereotaxic frame (Stoelting, Chicago, IL, USA). The bilateral PVN microinjections were carried out with two glass micropipettes (about 50 μm tip diameter) and completed within 1 min. Microinjection volume was 50 nl for each microinjection site. At the end of the experiment, same volume of Evans Blue (2%) was injected into the microinjection site for histological identification. Only the data from rats whose microinjection sites were within the boundaries of the PVN were used for analysis. Rats with microinjection sites outside the PVN or at the margin of PVN were excluded from data analysis. Total 14 rats in Sham and CHF groups were excluded from data analysis because the microinjection sites were not within the PVN (Figure 1). A representative coronal photograph of brain section which showed bilateral microinjection sites evaluated by 50 nl of Evans blue diffusion (upper panel) and a schematic representation of microinjection sites (lower panel) in the PVN of a rat were in Figure 1.

### PVN sample preparation

The rat was anaesthetized with an overdose of pentobarbital (100 mg/kg, i.p.). The brain of the rat was quickly removed, frozen in liquid nitrogen and stored at −80 °C until being used. Brain tissue was cut into 450 μm coronal section within the levels from 1.5 mm to 2.0 mm caudal from bregma with frozen microtome (Leica CM1900-1-1, Wetzlar, Hessen, Germany).

### Measurement of IMD and Ang II levels

PVN sample tissue was homogenized and the total protein in the homogenate supernatant was extracted and measured by using protein assay kit (BCA; Pierce). The IMD and Ang II levels in PVN tissue homogenate supernatant were measured using an enzyme-linked immunoassay kit (USCN Life Science Inc., USA). For both assays, the manufacturer's instructions were followed.

### Measurement of superoxide anions

The superoxide anions level in the PVN was detected using the lucigenin-derived chemiluminescence method [23]. Dark-adapted lucigenin (5 μM) reacts with superoxide anions and results in photon emission which can be measured with a luminometer (20/20n, Turner, CA, USA) once every minute for 10 min. The values were expressed as the MLU per minute per milligram of protein.

### Chemicals

IMD, AM22-52 and CGRP8-37 were obtained from Bachem (Hauptstrasse, Bubendorf, Switzerland), capsaicin, Ang II, tempol and lucigenin were purchased from Sigma Chemical (St. Louis, MO, USA).

### Experimental design

#### Experiment 1

IMD and Ang II levels in the PVN were determined with Elisa method in both Sham and CHF rats (n = 6 for each group).

#### Experiment 2

Effects of PVN microinjection of IMD (30 pmol) on the baseline RSNA, MAP and HR responses were investigated in Sham and CHF rats (n = 6 for each group).

#### Experiment 3

Effects of IMD, CGRP8-37, AM22-52 and Ang II on the CSAR were respectively investigated in Sham rats and CHF rats. The PVN microinjection of saline, two doses of IMD (3 or 30 pmol), CGRP8-37 (0.2 nmol), AM22-52 (1 nmol) or Ang II (0.3 nmol) were carried out in 6 groups of Sham rats and 6 groups of CHF rats (n = 6 for each group). To exclude the possibility that the effects of IMD were caused by diffusion to other brain area, the effects of microinjection of IMD (30 pmol) into the anterior hypothalamic area which is adjacent to the PVN were determined in CHF rats (n = 3).

#### Experiment 4

Effects of PVN pretreatment with saline, CGRP receptor antagonist CGRP8-37 (0.2 nmol) and adrenomedullin (AM) receptor antagonist AM22-52 (1 nmol) on the CSAR response to the PVN microinjection of IMD (30 pmol) were investigated in 6 groups of Sham rats and 6 groups of CHF rats (n = 6 for each group).

#### Experiment 5

Effects of PVN pretreatment with saline, IMD (30 pmol) and tempol (20 nmol) on the CSAR response to the PVN microinjection of Ang II (0.3 nmol) were investigated in 4 groups of Sham rats and 4 groups of CHF rats (n = 6 for each group). The PVN microinjection of Ang II was carried out 28 or 8 min after IMD or tempol pretreatment respectively, and CSAR was determined 2 min after Ang II microinjection.

#### Experiment 6

Effects of the PVN microinjection of saline, IMD (30 pmol) and Ang II (0.3 nmol) on the superoxide anions level in the PVN were investigated in 3 groups of Sham rats and 3 groups of CHF rats (n = 6 for each group).

#### Experiment 7

Effects of PVN pretreatment with saline, IMD (30 pmol) and tempol (20 nmol) on the superoxide anions level response to the PVN microinjection of Ang II (0.3 nmol) were investigated in 4 groups of Sham rats and 4 groups of CHF rats (n = 6 for each group).

### Statistics

Comparisons between two groups were made by Student's t test. One-way ANOVA followed by the Bonferroni test for post hoc analysis was used when multiple comparisons were made. The values were expressed as the mean±SE. P<0.05 was considered statistically significant.

## Results

### Anatomical and hemodynamic data

Mean infarct area was 33.4% of the LV in CHF rats, and there was no obvious infarct in Sham rats. Compared with Sham rats, heart weight, heart-to-body weight ratio and LVEDP were significantly increased in CHF rats, but LV peak systolic pressure (LVSP) and the maximum of the first differentiation of LV pressure (dp/dtmax) were markedly decreased in CHF rats (Table 1).

**Table 1:** Anatomic and hemodynamic data at the 8th week in Sham and CHF rats.

	Sham	CHF
Body weight (g)	367±10	359±8
Heart weight (mg)	1287±48	1546±54^*^
HW/BW (mg/g)	3.37±0.21	4.30±0.29^*^
MAP (mm Hg)	94.7±3.4	92.7±2.8
HR (beats/min)	388±7	396±6
Infarct size (% LV area)	0	33.4±2.9^*^
LVSP (mm Hg)	131±6	112±5^*^
LVEDP(mm Hg)	−2.08±0.28	20.8±2.0^*^
LV dp/dt_max_ (mm Hg/s)	3580±146	2062±102^*^

HW: heart weight; BW: body weight; MAP: mean arterial pressure; LV: left ventricle; LVSP: left ventricle peak systolic pressure; LVEDP: left ventricle end-diastolic pressure; LVdp/dt_max_: maximal rise rate of LV pressure. Values are mean±SE. *P<0.05 versus Sham. n = 7 for each group.

### CSAR

**Figure 2:**
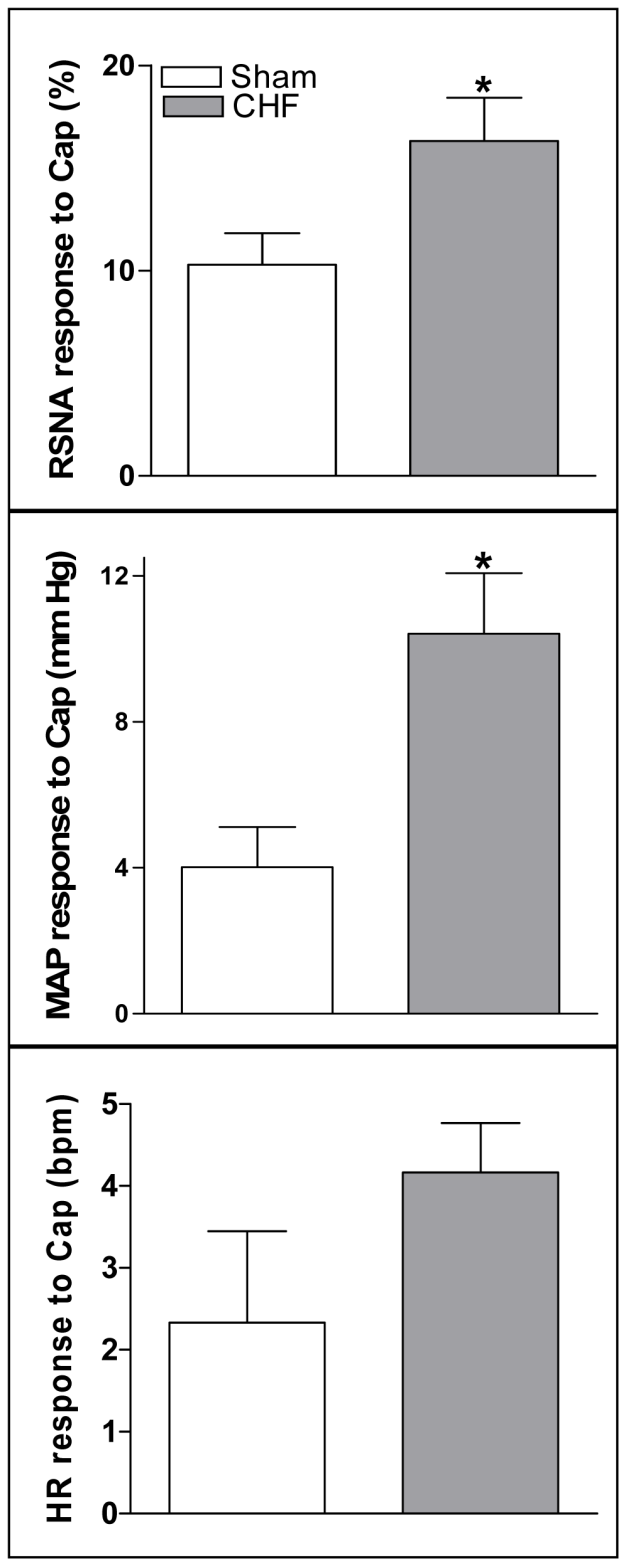
CSAR induced by epicardial application of capsaicin was evaluated by the RSNA and MAP responses to epicardial application of capsaicin (1 nmol). CSAR: cardiac sympathetic afferent reflex; RSNA: renal sympathetic nerve activity; MAP: mean arterial pressure; Cap: capsaicin. Values are mean±SE. *P<0.05 vs. Sham. n = 6 for each group.

In this study, the CSAR was induced by epicardial application of Cap and evaluated by the RSNA and MAP responses to Cap. The CSAR was significantly enhanced in CHF rats compared with Sham rats (Figure 2).

### IMD and Ang II levels in the PVN

**Figure 3:**
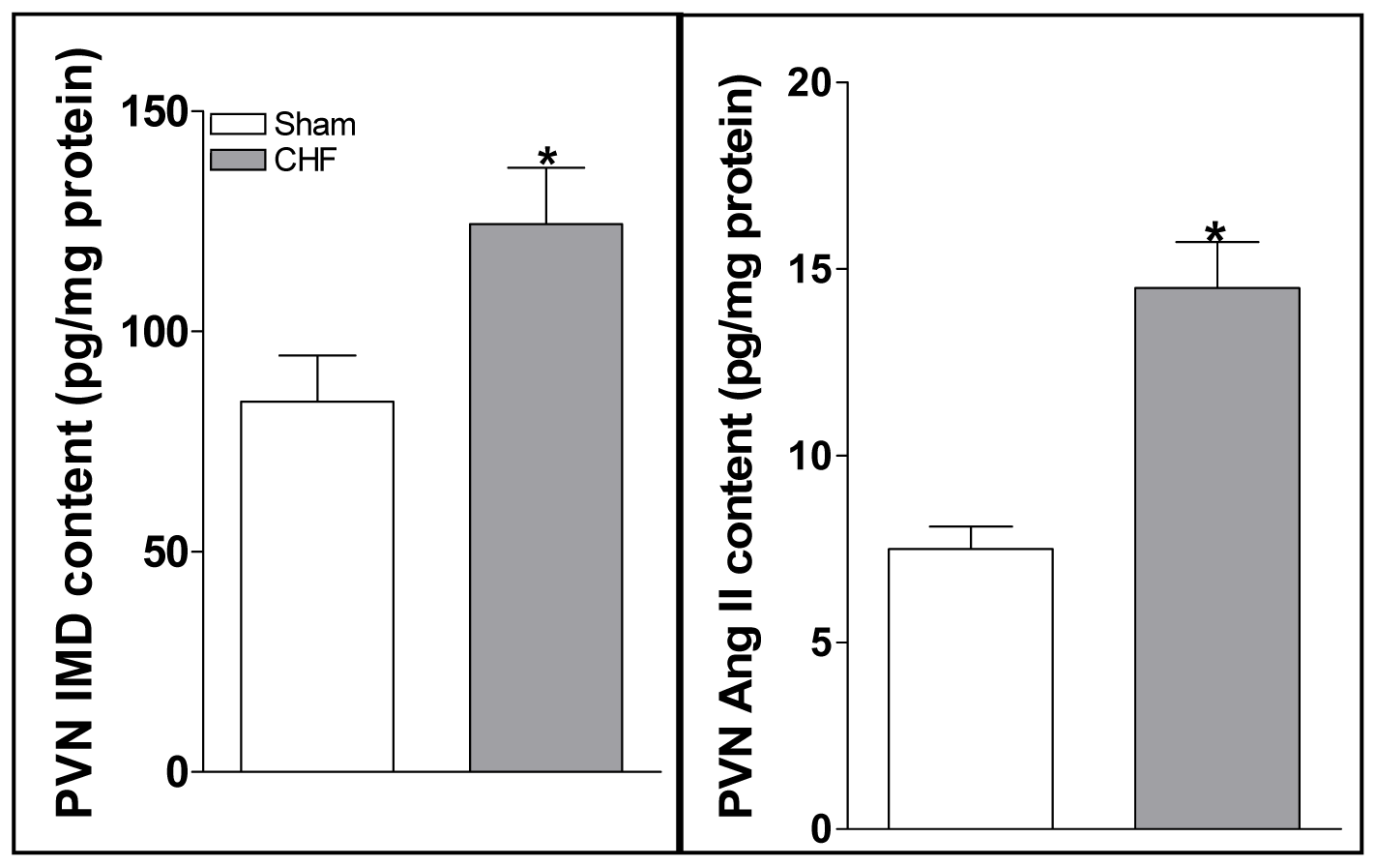
Levels of IMD and Ang II in the PVN in Sham rats and CHF rats. Values are mean±SE. *P<0.05 vs. Sham. n = 6 for each group.

IMD and Ang II levels in the PVN were much higher in CHF rats than Sham rats (Figure 3).

### Effects of IMD on the baseline RSNA and MAP and CSAR

**Figure 4:**
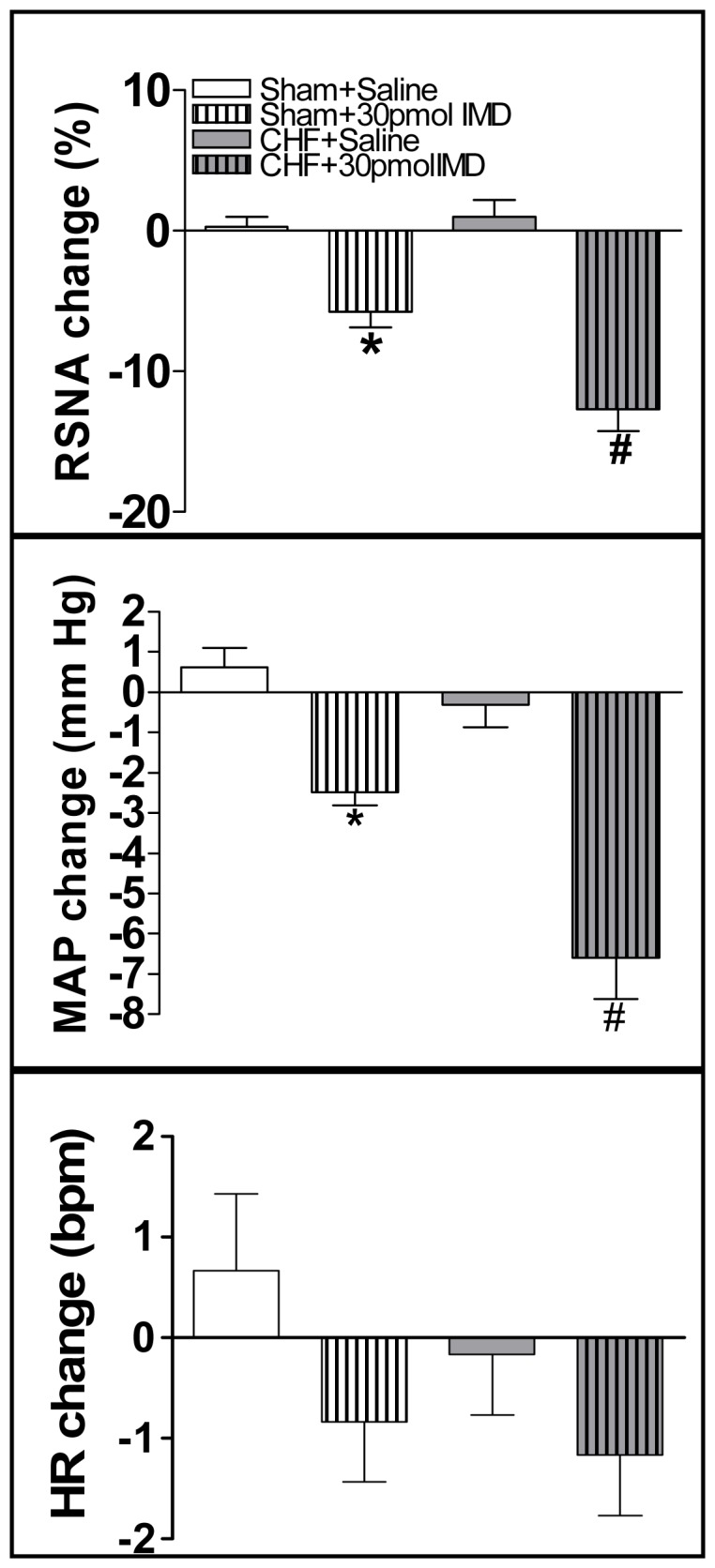
Effects of PVN microinjection of saline and IMD (30 pmol) on the baseline RSNA, MAP and HR in Sham rats and CHF rats. RSNA: renal sympathetic nerve activity; MAP: mean arterial pressure, HR: heart rate. Values are mean±SE. *P<0.05 vs. Saline; ^#^P<0.05 vs. Sham. n = 6 for each group.

**Figure 5:**
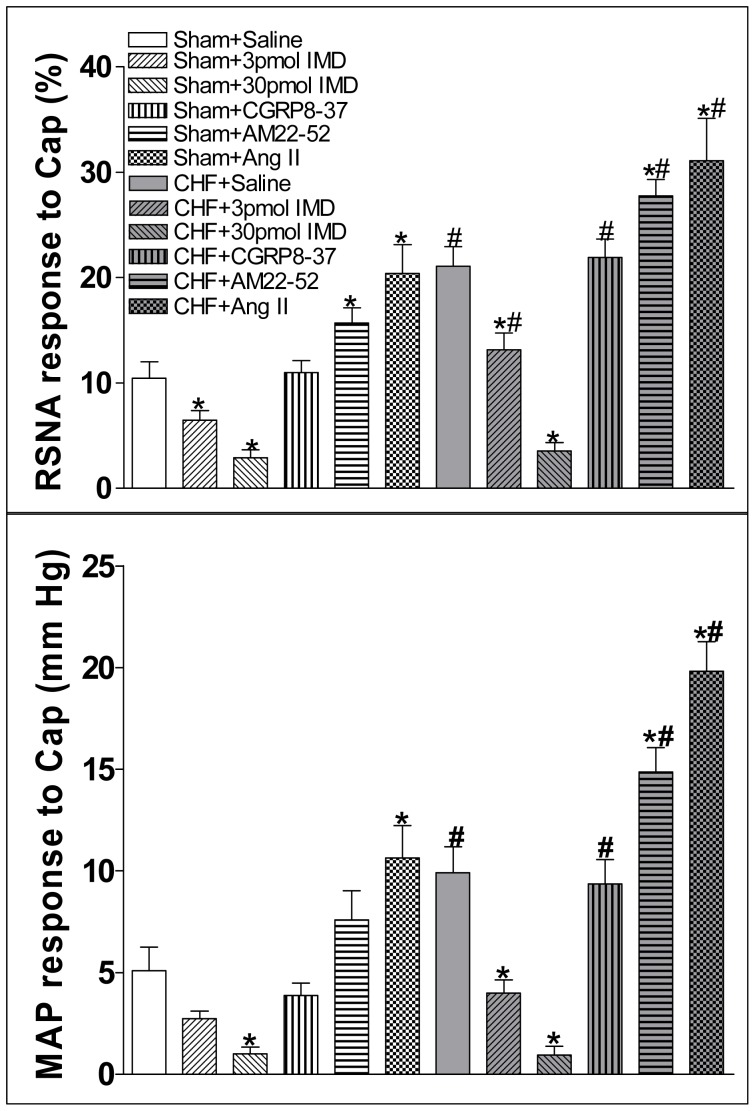
Effect of PVN microinjection of saline, two doses of IMD (3 or 30 pmol), CGRP receptor antagonist CGRP8-37 (0.2 nmol), AM receptor antagonist AM22-52 (1 nmol) or Ang II (0.3 nmol) on the CSAR. CSAR: cardiac sympathetic afferent reflex; RSNA: renal sympathetic nerve activity; MAP: mean arterial pressure; HR: heart rate; Cap: capsaicin. Values are mean±SE. *P<0.05 vs. Saline; ^#^P<0.05 vs. Sham. n = 6 for each group.

**Figure 6:**
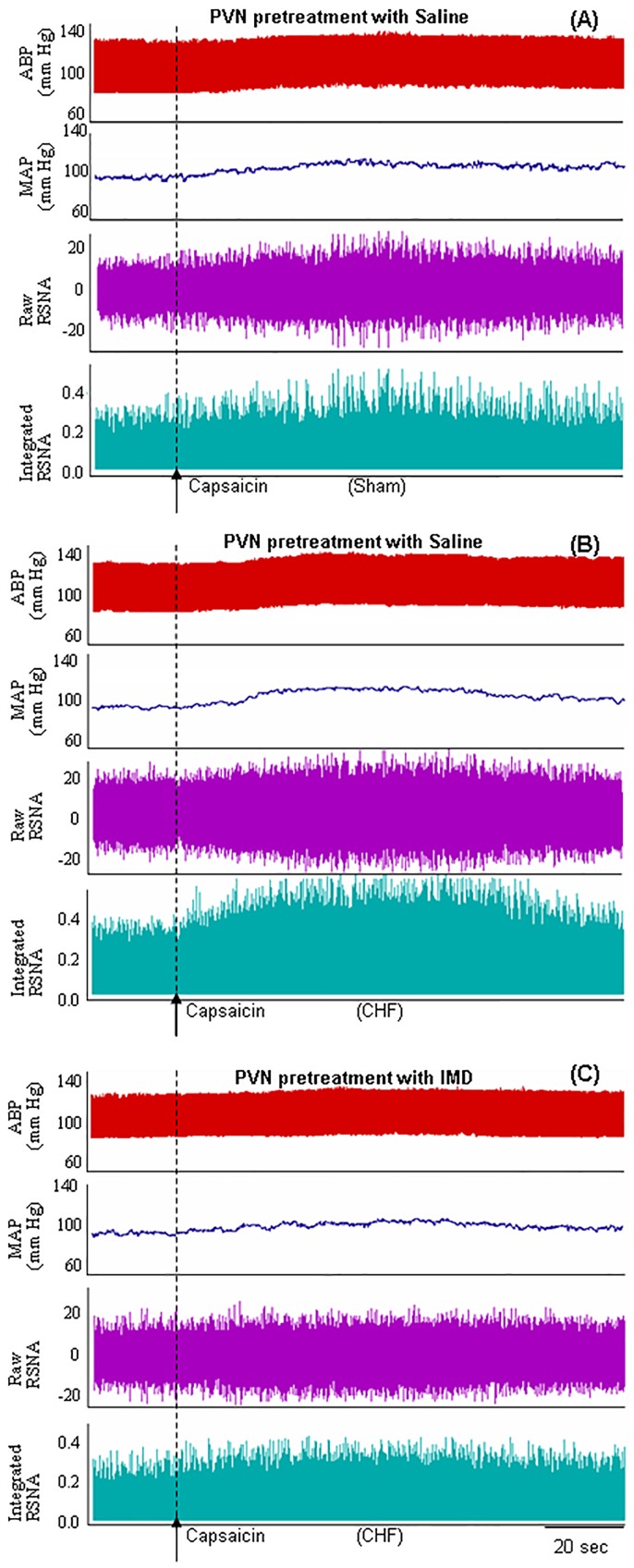
Tracing showing the effect of PVN microinjection of Saline in Sham and CHF rats and IMD (30 pmol) in CHF rats on CSAR. CSAR was induced by epicardial application of capsaicin. The CSAR in CHF rats (B) was stronger than Sham rats (A). IMD attenuated the enhanced CSAR in CHF rats (C). RSNA: renal sympathetic nerve activity; ABP: arterial blood pressure; MAP: mean arterial pressure.

IMD (30 pmol) significantly decreased the baseline RSNA and MAP in CHF rats compared with Sham rats (Figure 4). The effects of IMD on the baseline RSNA and MAP peaked at about 30 min and lasted at least 60 min. Microinjection of IMD (3 or 30 pmol) into the PVN caused greater decrease in the CSAR in Sham and CHF rats (Figure 5). IMD in the PVN had no effects on the baseline HR and HR response to Cap in Sham and CHF rats (Figure2, 4). Microinjection of IMD (30 pmol) into the anterior hypothalamic area, which is adjacent to the PVN, had no significant effects on the CSAR and the baseline RSNA and MAP in CHF rats (data not shown). The representative recordings showed that PVN microinjection of IMD (30 pmol) decreased CSAR in CHF rats (Figure 6).

### Effects of AM22-52 and CGRP8-37

**Figure 7:**
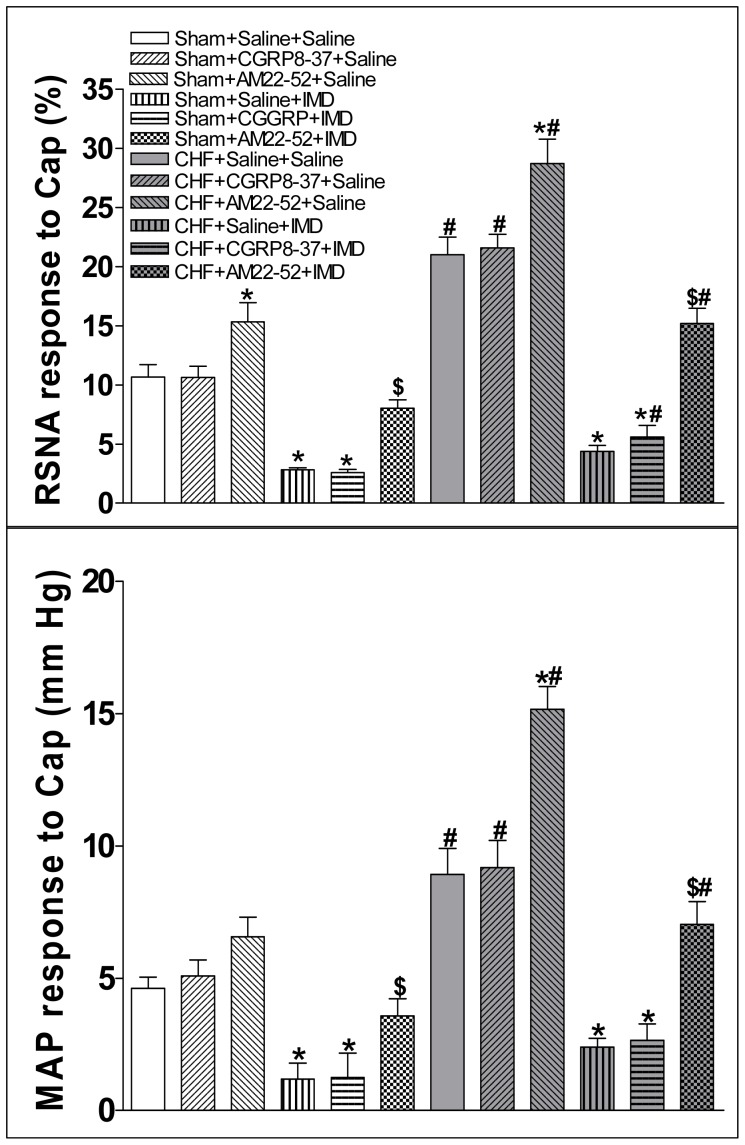
Effect of PVN pretreatment with saline, CGRP receptor antagonist CGRP8-37 (0.2 nmol) or AM receptor antagonist AM22-52 (1 nmol) on the CSAR response to the PVN microinjection of IMD (30 pmol). CSAR: cardiac sympathetic afferent reflex; RSNA: renal sympathetic nerve activity; MAP: mean arterial pressure; Cap: capsaicin. Values are mean±SE. *P<0.05 vs. Saline+Saline; ^$^P<0.05 vs. Saline+IMD; ^#^P<0.05 vs. Sham. n = 6 for each group.

AM22-52 microinjection into the PVN significantly increased the CSAR in both Sham and CHF rats but not CGRP8-37 (Figure 5). The reducement of CSAR response to IMD was inhibited by the pretreatment with AM22-52 (1 nmol) in the PVN in both Sham and CHF rats but not CGRP8-37 (Figure 7).

### Effect of IMD on Ang II-induced increase in CSAR

**Figure 8:**
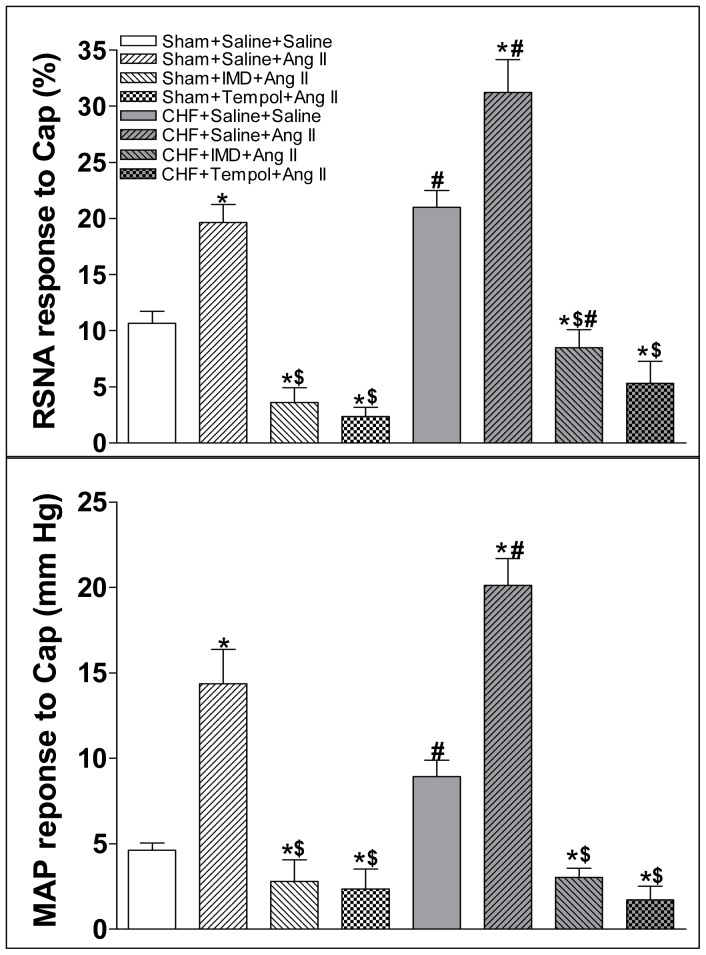
Effect of PVN pretreatment with saline, IMD (30 pmol) or tempo (20 nmol) on the CSAR response to the PVN microinjection of Ang II (0.3 nmol). CSAR: cardiac sympathetic afferent reflex; RSNA: renal sympathetic nerve activity; MAP: mean arterial pressure; Cap: capsaicin. Values are mean±SE. *P<0.05 vs. Saline+Saline; ^$^P<0.05 vs. Saline+Ang II; ^#^P<0.05 vs. Sham. n = 6 for each group.

Ang II (0.3 nmol) microinjection into the PVN significantly increased the CSAR in CHF rats compared with Sham rats (Figure 5). The augmented response of Ang II on CSAR was inhibited by the pretreatment with IMD (30 pmol) or tempol (20 nmol) in the PVN in both Sham and CHF rats (Figure 8).

### Superoxide anions level in the PVN

**Figure 9:**
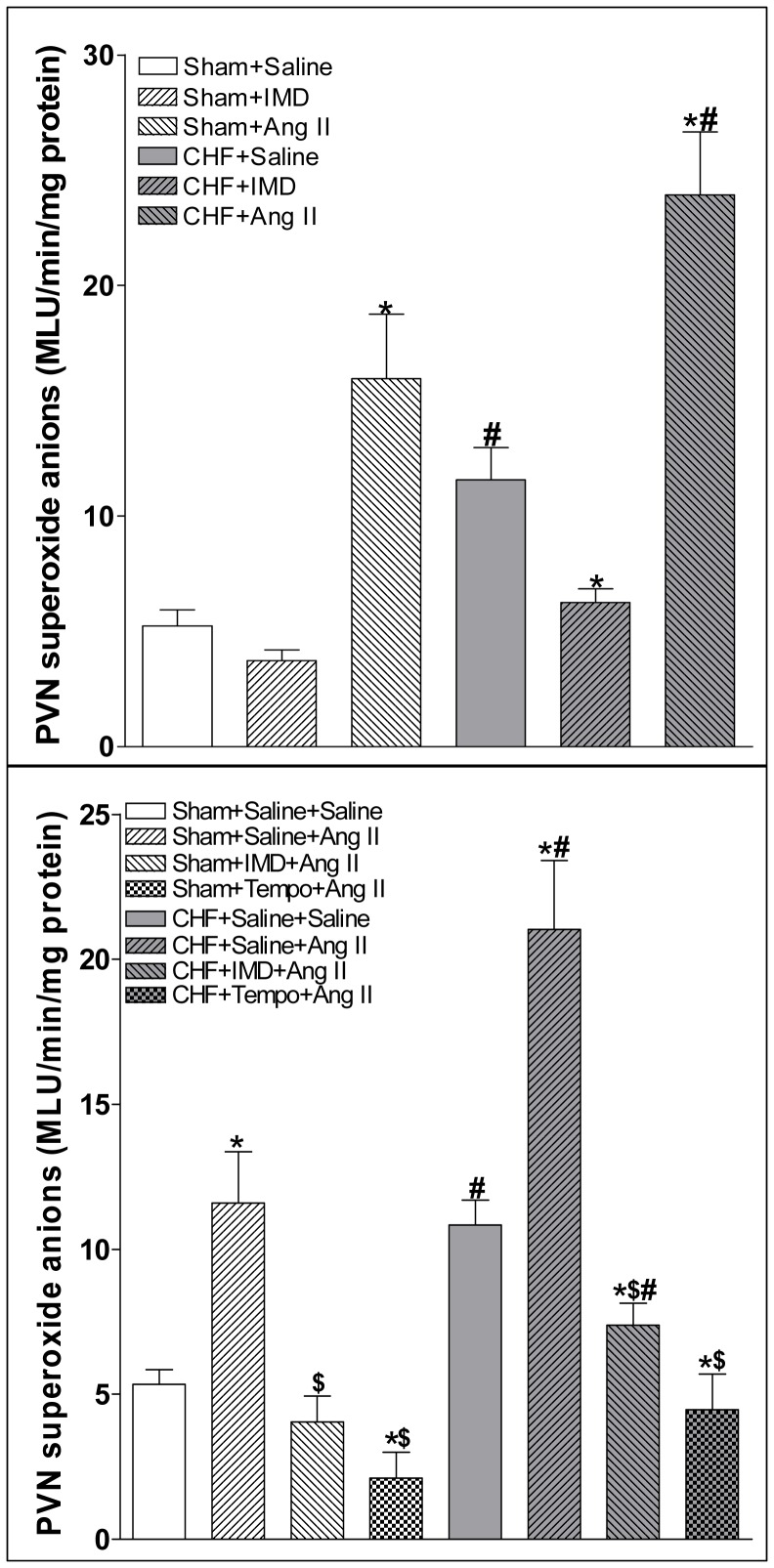
Effect of PVN microinjection of saline, IMD (30 pmol) or Ang II (0.3 nmol) on the superoxide anions level in the PVN and effect of PVN pretreatment with saline, IMD (30 pmol) or tempo (20 nmol) on the superoxide anions level caused by Ang II (0.3 nmol) in the PVN. Values are mean±SE. *P<0.05 vs. Saline; ^#^P<0.05 vs. Sham. n = 6 for each group (upper panel). Values are mean±SE. *P<0.05 vs. Saline+Saline; ^$^P <0.05 vs. Saline+Ang II; ^#^P<0.05 vs. Sham. n = 6 for each group (lower panel).

Microinjection of IMD (30 pmol) into the PVN significantly decreased the superoxide anions level in the PVN in CHF rats, but not in Sham rats (Figure 9). Microinjection of Ang II (0.3 nmol) into the PVN significantly increased the superoxide anions level in the PVN in Sham and CHF rats (Figure 9). PVN pretreatment with IMD (30 pmol) or tempol (20 nmol) significantly decreased Ang II-induced increase in superoxide anions level in the PVN in both Sham and CHF rats (Figure 9).

## Discussion

The primary findings in this study were that the levels of IMD and Ang II in the PVN were increased in CHF rats; IMD in the PVN significantly lowered the CSAR and the baseline RSNA and MAP in CHF rats than those in Sham rats; PVN pretreatment with IMD suppressed the enhanced CSAR and superoxide anions responses to Ang II in the PVN in Sham and CHF rats. AM receptor antagonist AM22-52 increased the CSAR and inhibited the effect of IMD on CSAR in Sham and CHF rats. These data indicate that exogenous IMD in the PVN inhibits CSAR via AM receptor in CHF rats, and attenuates the effect of Ang II in the PVN on CSAR, which may be through inhibiting Ang II-induced superoxide anions increase in the PVN.

IMD shares the receptor system consisting of CRLR and RAMPs with CGRP and AM [10]. In our recent study, all the receptor components were expressed in the PVN [30], CRLR, RAMP2 and RAMP3 protein expressions are apparent in the PVN, but the RAMP1 mRNA and protein expressions are low. In this study, the effect of IMD on CSAR was blocked with AM receptor antagonist AM22-52 but not CGRP receptor antagonist CGRP8-37, one possibility is that the level of RAMP1 expression remained small in CHF state and it was not enough to block the effect of IMD, the other is that IMD displayed its action on the CSAR through AM receptor not CGRP receptor. Furthermore, AM22-52 increased the CSAR in both sham rats and CHF rats, whereas CGRP8-37 had no significant effect. These results suggest that the AM receptor (CRLR/RAMP2 or CRLR/RAMP3) rather than CGRP receptor (CRLR/RAMP1) is involved in the CSAR in sham and CHF rats. The endogenous IMD in the PVN may have inhibitory effect on the CSAR via AM receptor in CHF. It is speculated that the increased IMD or AM receptor (CRLR/RAMP2 or CRLR/RAMP3) expression by various strategies in the PVN may be beneficial for the attenuation of CSAR. The higher level of IMD may be an important compensatory change in the PVN in CHF rats, which may contribute to the long-lasting sympathetic inhibition by attenuating the CSAR in CHF. In the future study, we should investigate whether endogenous AM could also inhibit the enhanced CSAR by binding with its receptor complex (CRLR/RAMP2 or CRLR/RAMP3) in the PVN in CHF rats and whether the effect of IMD on CSAR is associated with unidentfied receptor in the PVN.

Ang II and superoxide anions in the PVN causes exaggerated increases in SNA and CSAR in rats with CHF [23], [25], [42], and Ang II in the PVN promotes the increase of superoxide anions which can strengthen its effect on the CSAR in CHF rats [23]. AM-knockout mice showed Ang II/salt-loading-induced cardiovascular injury, all associated with enhanced ROS generation [43]. IMD is known to inhibit rat cardiac fibroblast activation induced by angiotensin II [44], and it exerts an antihypertrophic effect caused by Ang II on neonatal cardiomyocytes by reducing the level of superoxide [34]. These results suggest that IMD may have an inhibitory effect on Ang II-induced CSAR response by lowing superoxide anions level. In this study, the levels of Ang II and superoxide anions in the PVN were increased in CHF rats, IMD in the PVN decreased superoxide anions level and attenuated Ang II-induced CSAR response and superoxide anions increase in the PVN in CHF rats, which indicate that the inhibitory effect of IMD on CSAR is partially through inhibiting Ang II-induced superoxide anions.

The level of IMD is augmented in the presence of oxidative stress in hypertrophied cardiomyocytes [45]. IMD has a potential protective role against oxidative stress in human aortic endothelial cells [46] and rat cerebral endothelial cells [40]. It ameliorated vascular and renal injury in DOCA-salt hypertensive rats [39] and attenuated chemia/reperfusion-induced myocardial injury by inhibiting oxidative stress [37]. NAD(P)H oxidase in the PVN is a major source of the ROS in modulating the CSAR, and Ang II microinjecrion into the PVN significantly increases NAD(P)H oxidase activity which contributes to the effect of Ang II on CSAR [47]. The inhibition of NADPH oxidase involves in the effects of IMD on attenuating myocardial oxidative stress injury induced by ischemia/reperfusion [37] and ventricular myocyte hypertrophy induced by Ang II in neonatal rat [34]. These findings suggest that the inhibition of NADPH oxidase may be one of the mechanisms of IMD in decreasing superoxide anions in the PVN in CHF rats or attenuating Ang II-induced superoxide anions in the PVN, which needs to be explored in future study.

The enhanced CSAR is involved in the sympathetic over-activation in CHF, and superoxide anions and Ang II in the PVN promote the increases in SNA and CSAR in CHF rats. In the present study, we found that IMD and Ang II in the PVN were increased in CHF rats; IMD in the PVN inhibited SNA and attenuated the CSAR, and blockade of AM receptors in the PVN partially prevented its effect; PVN IMD decreased superoxide anions and attenuated the effects of Ang II on CSAR and superoxide anions level in the PVN in CHF rats. These results indicate that IMD inhibits SNA in CHF rats, which is through attenuating the enhanced CSAR response to superoxide anions and Ang II in the PVN. Activation of AM receptors in the PVN mediates the IMD's effects and IMD in the PVN is an important mechanism in attenuating CSAR and sympathetic activation in CHF.
